# Translatability and transferability of in silico models: Context of use switching to predict the effects of environmental chemicals on the immune system

**DOI:** 10.1016/j.csbj.2022.03.024

**Published:** 2022-03-26

**Authors:** Francesco Pappalardo, Giulia Russo, Emanuela Corsini, Alicia Paini, Andrew Worth

**Affiliations:** a Department of Health and Drug Sciences, Università degli Studi di Catania, Italy; b Department of Pharmacological and Biomolecular Sciences, Università degli Studi di Milano, Italy; c European Commission, Joint Research Centre (JRC), Ispra, Italy

**Keywords:** In silico trials, Immune system, PFAS, Risk-assessment, Vaccines, Immunotoxicity

## Abstract

Immunotoxicity hazard identification of chemicals aims to evaluate the potential for unintended effects of chemical exposure on the immune system. Perfluorinated alkylate substances (PFAS), such as perfluorooctane sulfonate (PFOS) and perfluorooctanoic acid (PFOA), are persistent, globally disseminated environmental contaminants known to be immunotoxic. Elevated PFAS exposure is associated with lower antibody responses to vaccinations in children and in adults. In addition, some studies have reported a correlation between PFAS levels in the body and lower resistance to disease, in other words an increased risk of infections or cancers.

In this context, modelling and simulation platforms could be used to simulate the human immune system with the aim to evaluate the adverse effects that immunotoxicants may have.

Here, we show the conditions under which a mathematical model developed for one purpose and application (e.g., in the pharmaceutical domain) can be successfully translated and transferred to another (e.g., in the chemicals domain) without undergoing significant adaptation. In particular, we demonstrate that the Universal Immune System Simulator was able to simulate the effects of PFAS on the immune system, introducing entities and new interactions that are biologically involved in the phenomenon. This also revealed a potentially exploitable pathway for assessing immunotoxicity through a computational model.

## Graphical abstract

**Figure:**
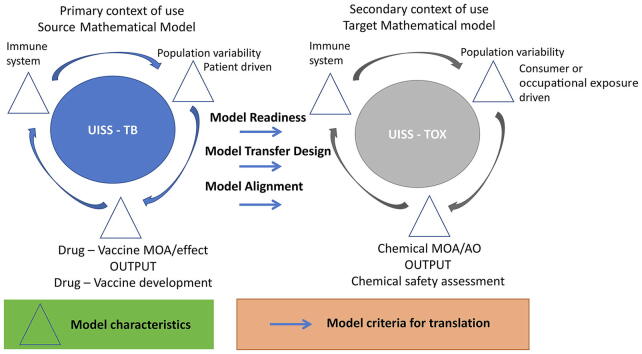

## 1 Introduction

The immune system has evolved to protect us against harmful substances, germs and transformed cells, thereby preserving the integrity of the body. Immune cells are an integral part of many systems, including the respiratory, dermal, gastrointestinal, neurological, cardiovascular, reproductive, and endocrine systems [1]. As a consequence, exposure to immunotoxic compounds can have serious adverse health consequences affecting responses to both communicable and non-communicable diseases [2].

Immunotoxicology is the study of immune system dysfunction that can result from exposure to a variety of chemicals or biologic agents that alter the immune system, resulting in an adverse effect for the host, which range from reduced resistance to infection and neoplasia to allergic and autoimmune conditions. Immunotoxic compounds can alter the number of cells (innate or adaptive), the ability of the cells to produce cytokines, chemokines, antibodies or growth factors, the composition of cell subpopulations occupying the site of response, or the function of cells (i.e., killing of the infected cells or cell proliferation). This could lead to an increased incidence in infections or tumour burden. The potential for exposure to immunotoxic compounds poses a serious concern for the public as well as regulatory agencies. It is therefore important to understand the immunotoxic potential of xenobiotics and the risk they pose to humans [3].

Decades of research has resulted in the development of specific animal assays and the identification of sensitive endpoints that measure effects on the immune response [4], [5], on the basis of which many regulatory agencies have developed specific immunotoxicity testing guidelines [6], [7].

Currently, the assessment of chemical immunotoxicity relies mainly on animal models [7]. However, in recent decades considerable progress has been made, and several in vitro methods have been validated to assess inappropriate immunostimulation. While the main achevements in using in vitro models to assess immunotoxicity have focused on chemical sensitization, and in particular, on skin sensitization [8], [9], important progress has also been made in the identification of immunosuppressive compounds [10], [11], [12]. Considering the complexity of the immune system, it is likely that several in vitro assays will be needed to identify immunotoxicants, and a tiered approach is believed to be the most appropriate means to assess immunotoxicity in vitro [13].

Any alteration in immune function (e.g., antigen presentation, cytokine production, cell proliferation) that significantly deviates from control values and can be linked to a downstream immunotoxic effect (i.e., immunosuppression, hypersensitivity, autoimmunity) should be considered as an adversity. Several isolated processes can be studied in vitro including antigen presentation, lymphocyte proliferation, cytokine production, phagocytosis, lysis, and even primary antibody production, offering the possibility to assess immunotoxicity in vitro*.* In the future, based on the considerable progress in 3D models with engineered immune tissues and organs, we may foresee that it will be possible to identify any direct immunotoxic substance in an integrated model of the whole human immune system [14]. Currently, we have to rely on a combination of different tests.

Per- and polyfluoroalkyl substances (PFAS), such as perfluooctanoic acid (PFOA) and perfluorooctane sulfonic acid (PFOS), are persistent, globally disseminated environmental contaminants. They possess a strong carbon–fluorine bond, which leads to their environmental persistence. The presence in the molecule of both hydrophilic and hydrophobic portions makes these compounds useful as surfactants and dispersants, and PFAS have been used extensively in many commercial and industrial applications for the last 70 years [15]. More than 200 use categories and subcategories have been identified for more than 1400 individual PFAS [15]. The Organisation for Economic Co-operation and Development’s chemical inventory reports over 4000 substances that contain at least one perfluoroalkyl moiety^6^.

Due to their widespread use and environmental persistence, PFAS are an important class of environmental contaminants and are of major toxicological concern [16], [17]. They are found in water, air, fish, and soil at locations across the globe, with concentrations of PFAS in surface and groundwater ranging in value along the ng/L-μg/L scale [16]. Moreover, exposure to PFAS has been linked to harmful health effects in humans and animals (EFSA Opinion, 2020). PFAS are widespread despite some being phased out, and have been detected in different continents irrespective of the level of industrialization, indicating long-range atmospheric transport as an important pathway of PFAS distribution [16].

Epidemiological studies have shown associations between exposure to specific PFAS and a variety of health effects, including altered immune and thyroid function, lipid and insulin dysregulation, liver disease, kidney disease, reproductive and developmental toxicity, and cancer [17]. Based on studies in animals and humans, effects on the immune system have been considered by EFSA the most critical for the risk assessment, with effects often observed at lower exposure levels than those causing effects on the liver and thyroid hormones [18]. There is evidence from both epidemiology and laboratory studies that PFAS are immunotoxic, affecting both cell-mediated and humoral immunity [19], [20], [21]. Overall in humans, the evidence of PFAS immunosuppression shows strong evidence of diminished vaccine efficacy, some indications of increased risk of infections, and limited indication of allergies, asthma and atopic dermatitis following *in utero*, infant, and early childhood PFAS exposures [22], [23].

In laboratory animals, reported effects of PFAS in laboratory animals include decreased spleen and thymus weights and cellularity, altered cytokine production, reduced specific antibody production, and reduced survival after influenza infection. Elevated PFAS blood levels are associated with lower antibody responses to vaccinations in children [24], [25], [26], [27] and in adults [28]. In addition, some studies reported a correlation between PFAS levels in the body and lower resistance to disease, in other words an increased risk of infections or cancers [29], [30], [31]. A relationship between higher PFAS levels and increased risk of asthma as well as increased adolescent food allergies have been reported in some studies [32], [33], but overall the evidence is limited [22], [23].

Regarding the underlying mechanisms, many PFAS are ligands of the nuclear peroxisome proliferator-activated receptors (PPAR), with different kinetics, patterns and potency among species [34]. These receptors regulate lipid homeostasis, inflammation, adipogenesis, reproduction, wound healing, and carcinogenesis [35]. Binding to PPARs results in the modulation of the transcription of downstream genes containing the peroxisome proliferator response element, which leads to altered expression of genes including those related to metabolism of sex steroids and thus leading to abnormal physiological function of sex steroids [36]. In addition, PFAS have been shown to interact with receptors and transcription factors other than PPARα, including PPARγ, CAR (constitutive activated/androstane receptor), estrogen receptor alpha (ERα), androgen receptor, glucocorticoid receptor, pregnane X receptor, the transcription factor Nrf2 (nuclear factor erythroid 2-related factor 2), and NF-kB [17]. All these are central in immune cell activation, and their modulation by PFAS provides a biological plausible link to the adverse effects observed. Reduced antibody production has been clearly associated with PFAS exposure. Multiple cell types are involved in the T cell dependent antibody response. Initially, the antigen is recognized and presented by antigen presenting cells in a MHC class II mediated mechanism to naive T cells, and activation of B cells by T cells with antibody formation, with cross talk between all involved cell types using receptor/ligand and cytokine interactions. In experiments with selected PFAS, namely PFOA, PFOS, PFBS, PFOSA, PFDA, and fluorotelomer, we have observed different effects on LPS and PHA-induced cytokine production (i.e., IL-6, IL-8, TNF-α, IL-4, IL-10 and IFN-γ) [37]. Our results indicate that PFOA is the least active of the PFAS examined followed by PFBS, PFDA, PFOS, PFOSA and fluorotelomer. Leukocytes obtained from female donors appear to be more sensitive to the in vitro immunotoxic effects of PFCs when their responses are compared to the results obtained using leukocytes from male donors. Mechanistic investigations demonstrated that inhibition of TNF-α release occurred at the transcriptional level. All PFAS tested decreased LPS-induced NF-κB activation, while, with the exception of PFOA, none of the PFAS tested was able to activate PPARα driven transcription in transiently transfected human promyelocytic THP-1 cells, indicating that PFAS directly suppress cytokine secretion by immune cells, with different mechanisms of action. Most of the toxicity data available are for a handful of PFAS, mainly legacy PFAS such as PFOA and PFOA. It is, therefore, clear that information on modes of action and adverse outcome pathways must be expanded. Considering the profound differences in PFAS toxicokinetic properties, additional studies are necessary for a proper understanding of differences in responses between the sexes and among species and life stages.

In the current study, PFOA and PFOS have been selected as reference PFAS as they are two of the most widely used and studied chemicals in the PFAS group. As mentioned above, several studies document that PFAS exposure is associated with suppression in at least one measure of the anti-vaccine antibody response with evidence from developmental, childhood, and adult exposures [22]. The response to vaccination was therefore chosen as the immunological parameter. This was also identified as the critical parameter for risk assessment by EFSA (EFSA opinion, 2020).

Considering the hundreds of PFAS used, alternative approaches to animals are essential to investigate their immunotoxicity. Alongside animal models, ex vivo methods and in vitro methods, immunotoxicology should take advantage of the progress made in computational immunology. In this regard, the Universal Immune System Simulator (UISS) may offer the opportunity to estimate the immunotoxicity risk posed by immunotoxicants. The UISS is a mechanistic computational platform that simulates the human immune system, providing the possibility to investigate the effects on vulnerable populations, like children and elderly people. Nowadays, the possibility of replacing in vivo experiments with in vitro methods and computer simulations is no longer a chimera, providing the opportunity to establish new knowledge, and approaches to protect health from potential immunotoxic compounds. The UISS belongs to In Silico Trials (IST) modelling and simulation. An IST refers to ‘the use of individualized computer simulation in the development or regulatory evaluation of a medicinal product or medical device/medical intervention’ [38]. Different modelling and simulation approaches applied to IST can span from purely computer science techniques, such as agent-based modelling (ABM) and machine learning (ML), to mathematical approaches (i.e., differential equations, finite elements and regression analyses). The ABM paradigm is built on simulating the dynamics of single interacting entities according to well-known principles. This modelling technique thus captures emerging global complex behaviour from local interactions. ABM has been widely applied in a variety of biomedical scenarios, including precision medicine [39], [40], 2D and 3D experimental cultures [41], [42] and vaccine development and immune system response prediction [43], [44]. Because ABM makes it simple to create individualized models, its application to IST may be thought of as simulating virtual patients who can help attain the numbers required for statistical significance. Furthermore, the method can anticipate medication effects in persons who are not typically included in clinical studies (young and elderly patients or those affected by immune system dysregulation). The UISS offers a more modern and high-throughput approach, with the potential to accelerate the gathering of toxicity information on emerging and legacy PFAS. The UISS has been applied in several contexts of use, ranging from disease dynamics assessment, medicinal product outcome predictions, and dosage optimization. Recently, the UISS was used as an in silico trial platform for predicting the immune system response elicited by a therapeutic vaccine against tuberculosis (UISS-TB) [45], [46].

The idea behind this study was that a mathematical model, extensively used in support of a policy and built to answer a decision-making challenge, can be transferred to support resources in areas where data are deficient, and modelling built for one scope could be potentially applied to fill in the gaps. Hence, with the following strategy approach, we try to maximise this transition by the application of UISS in silico solution to immunotoxicology. Reusing by repurposing what is already available represents an efficient use of resources, reducing the need for testing, and saving time in the development and evaluation of the mathematical model.

The present work describes criteria for model repurposing. This is not the first time that a specific mathematical model developed in one field has been reused in another. However (to the best of our knowledge), it is the first time that precise criteria have been suggested to guide model repurposing. Noteworthy is the example of Physiologically Based Kinetic (PBK) models. These models are mathematical descriptions of the body that consider exposure, absorption, distribution, metabolism, and excretion of a chemical. They have been developed for more than 50 years in pharmacology (first reporting of such models dates to 1937). They are widely used in drug development and for drug dossier submissions [47]. In recent decades, these models have been explored for their applicability in chemical risk assessment. The use of these models in this domain is mainly to support hazard characterization and fill in data gaps without animal testing.

**Fig. 1:**
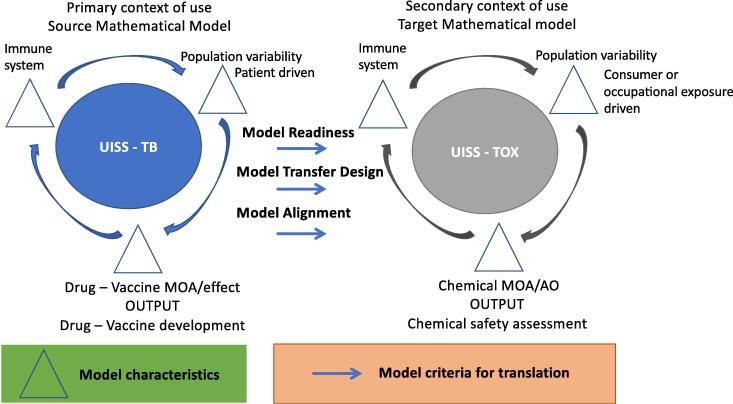
Schematic representation of a proposed strategy for evaluation of Model Translatability and Transferability of health models.

The first step in repurposing a mathematical model is to understand the context of use; it is essential to have information from both sides (primary/source use versus secondary/target) to identify similarities and differences. This should be done by identifying the model characteristics that drive the model development, characterisation and performance. Identification of these characteristics is essential for model transfer from one domain to another. These characteristics will have their challenges and limitations, which should be reported, such as model complexity, data quality, and variability. In particular, for a model to be transferable a different setting, the following three criteria need to be fulfilled (Fig. 1):

**Criterion 1. Model Readiness** (scientific component with motivation to transfer, intent to use, goals, knowledge). UISS-TB already contains all the entities, interactions, functions and hallmarks of the human immune system. Physiology, disease and treatment modelling and simulation layers deal with tuberculosis. So, from the point of view of readiness, UISS-TB is ready to be switched to another context of use dealing with other external insults like environmental chemicals that affect the immune system functionality (e.g., modified vaccine response in the presence of environmental chemicals).

**Criterion 2. Model Transfer Design** (application & goal settings practice, modelling application, and review of valid credible content). Keeping the core of immune system implementation in UISS, only the implementation of mechanisms of action of immunotoxicants is needed for the model transfer design.

**Criterion 3. Model Alignment** (support for basic understanding, support for decision making, competence & verification/qualification/validation context, and use dissemination). The Universal Immune System Simulator for the evaluation of immunotoxicants effects (UISS-TOX) can be used as a non-human expert risk assessor for evaluating the impact of immunotoxicants on the immune system function. In addition. components that are already validated and qualified in terms of immune system modelling core, do not need to undergo a revalidation/requalification process.

The aims of the present paper are the following: 1. To develop a module as part of the UISS to predict immune system perturbations after exposure to chemicals (UISS-TOX). 2. To simulate the class of PFAS as a case study. 3. Repurposing to understand the capacity when a mathematical model developed for one purpose is translated and transferred from one “language” (application) to another without undergoing fundamental change. So that if the mathematical model is valid in the field developed it can be applied to another one.

## 2 Software and methods

In computer science, Agent-Based Models (ABMs) are a simulation approach in which entities are followed individually, as opposed to differential equation systems [48], [49]. Complex emergent behaviour can form in this fashion, leading to the prediction of non-coded dynamics [50]. The entities, referred to as “agents,” are often arranged in a simulation space (i.e., a lattice), with several entities per lattice point [51]. Such agents can be heterogeneous, have internal features (such as longevity, internal state, and energy), and behave independently or as a result of interactions with other agents (e.g., move, interact with other agents in their neighbourhood, alter their internal state, or die) [52]. ABMs provide a number of benefits. They can be stochastic by definition and incorporate both delays and a spatial description in their behaviour. Furthermore, they provide a more accurate description of the biological characteristics and behaviour of the entities involved [53]. As a result, it is the biological information, rather than the modelling methodology, that is frequently the limiting factor in the correctness of the model description. Nonlinear behaviour, as well as the ability to add more complexity and biological knowledge, are not obstacles to solving the model [54]. Because such methods are numerically stable as integer values and reflect the majority of the variables involved, only a few complex floating-point operations are necessary.

### 2.1 Universal immune system simulator

To assess the immunotoxicity of PFAS, we used the Universal Immune System Simulator (UISS), a computer modelling and simulation platform that can replicate the major characteristics and dynamics of immune system activities. It is based on the agent-based model paradigm, which was created to reflect the immune system's response to general pathogens [44], [45], [55]. UISS is developed fully in the ANSI C-99 standard programming language, allowing us to create a platform that is architecture-independent. Both cellular and molecular entities are considered in UISS. Typically, cellular entities are studied separately and modelled as single agents. Position, half-life, and an internal state from a certain set of acceptable states are all characteristics of cell agents. State changes are used to realize their dynamics. Instead, the concentration of molecules per lattice-site is examined.

When a cell agent interacts with another agent, such as a cell, a molecule, or both, a state shift occurs. The immune system's most significant cells are B lymphocytes, helper, cytotoxic, and regulatory T lymphocytes, and natural killer cells [56]. Monocytes are also present, as well as macrophages and dendritic cells. B and T lymphocytes, for example, have particular receptors for modelling specificity [57], [58], just like their real-life counterparts. The model distinguishes between simple low molecular weight molecules like interleukins or signalling molecules in general [59] and more complicated molecules like immunoglobulins and antigens [60], for which specificity must be represented. Immune system activities are represented at the same level as entities. Both interactions and functions are included.

Central immune system's functions are referred to as functions. The UISS is particularly concerned with the diversity of specific elements, restriction of major histocompatibility classes [61], clonal selection by antigen affinity [62], thymus education of T cells [63], antigen processing and presentation (both the cytosolic and endocytic pathways are used) [64], cell–cell cooperation [65], homeostasis of bone marrow-derived cells [66], hypermutation of antibodies [67], cellular and humoral responses [68], and immune memory [69]. Time is discrete in UISS, as it is in most ABM techniques [70]. This means that all system actions are monitored and measured using time intervals that are evenly separated. All immune system activities, including interaction and diffusion processes, are maintained at each time interval.

An interaction between two entities is a complex action that eventually results in one or both entities' states changing. To interact, the entities must be “close enough.” The concept of the lattice-site is used to model physical proximity in more detail [71]. All interactions between cells and molecules occur in a single time step within a lattice-site, hence there is no correlation between entities residing at various sites at the same moment. Depending on the task at hand, the simulation space in UISS can be represented as a 2D L × L hexagonal lattice (six neighbours) or as a 3D L × L × L cubic lattice, with periodic boundary conditions or hard walls on the edges. Having the same diffusion coefficient, all entities are permitted to travel with a uniform probability between neighbouring lattices in the grid (Brownian motion) [72].

This simulation area is used to depict three anatomical compartments: the thymus, bone marrow, and a component of a generic secondary organ, from a biological rather than a physical standpoint. Interactions can be thought of as Bernoulli events [73], with each interaction having a probability of p. Aspecific and specific interactions are two types of interactions. When we consider Toll-like receptors (TLRs), for example, we know that they identify pathogen-associated molecular patterns (PAMPs) expressed by pathogens with limited specificity [74]. These will not be explicitly represented in UISS; instead, for all interactions involving the same couple TLR-PAMP, a fixed probability p' will be employed. Cells from adaptive immunity that are equipped with unique receptors are involved in distinct reactions.

Specific interactions necessitate a period of recognition between the two entities; in this situation, the probability p of contact is determined by the outcome of the recognition phase, in which the affinity between the implicated receptors plays a significant role. UISS models affinity by representing receptors and ligands as binary strings and using a string-matching mechanism [75]. Farmer and Packard [76] devised a simple technique to duplicate the conventional chemical complementarity mechanism between receptors. While this may appear to be a crude simulation of the true biological event, millions of recognitions can be processed quickly, allowing researchers to examine immune system features on a massive scale. Furthermore, when models based on this approach were benchmarked to experiment, they produced accurate results, indicating that the abstraction captures essential features of receptor/ligand binding and is not a limiting factor for the study of many biological scenarios [77].

Using the Hamming distance [78], the string-matching procedure's binding rule simulates the complementarity process between two receptors. The number of mismatched bits between two strings is measured by this distance. As a result, repertoires are represented as sets of strings in the model, and the set of lymphocyte receptors is represented as bit-strings of length h, forming the so-called “shape space.” The same clonotypic receptor, i.e., the same bit-string of length l, characterizes a clonal set of cells, and the potential repertory of receptors scales as 2 ^l^. The UISS is thus defined as a polyclonal bit-string lattice technique. Polyclonal refers to the ability to have many clones of lymphocytes of varied specificity, while bit-string refers to the fact that molecules and specificity among molecules are represented.

Finally, lattice denotes that the space is represented by a discrete lattice. Haematopoiesis and thymus selection are two of the most important mechanisms that control immune system activities. Haematopoiesis is a biological process that describes the production of blood cells from hematopoietic stem cells, for example. This process is included in the UISS to describe the creation of B and T lymphocytes in the “bone marrow compartment.” Furthermore, thymus selection holds a further selection of T helper cells (TH) and cytotoxic T cells in the “thymus compartment.” In the absence of perturbations, haematopoiesis is represented as an Ornstein–Uhlenbeck mean-reverting process [79] to keep the system in a metastable condition (cell homeostasis).

Thymus selection is utilized to ensure that a repertoire of MHC-restricted self-tolerant T cells is accessible in the UISS. The treatment employs a selective approach to imitate the actual biological events that occur during T cell maturation in the thymus. This process is made up of two sub-sequential stochastic procedures: a positive selection and a negative selection. T cells with limited affinity to MHC molecules alone (class I for cytotoxic T cells (TC) and class II for T helper cells (TH)) are eliminated in the initial phase because they are ineffective. Cells that pass the first selection phase are subjected to the second selection step, in which T cell receptors are compared to an MHC-self-peptide complex. If there is a high affinity between the two strings, such T cells are removed to prevent autoreactivity.

Of course, receptors, MHC molecules, and (self) peptides are always represented as binary strings, and the affinity is determined as explained above. Many more biological processes that are part of the immune system machinery are carried out. These include the Hayflick limit on cell duplication [80], immunological memory management [81], antibody hypermutation [82], bystander impact [83], isotype switching [84], anergic [85], antigen digestion and presentation [86], and B cell receptor mutation [87], [88].

An initialization step is carried out prior to the start of the simulation. During this phase, the lattice is filled with the required number of entities. The simulation is then run for a predetermined number of time-steps.

Both interaction-driven and non-interaction-driven activities (i.e., movement or internal processes) are completed at each time step. It should be highlighted that, in an ideal world, all processes within a time step should occur at the same moment. This is not a problem for movement or internal dynamics that can be easily replicated as serialized processes, but it could be a source of bias for interaction dynamics. In real computers, huge parallel execution is difficult to achieve [89] especially when the number of entities is large. For this purpose, a distinct random interaction scheme is constructed for each lattice-site, taking into account a random order of the interaction rules as well as a random order in the list of agents that may interact inside the same rule.

For a given rule that refers to two entity types A and B, every entity of type A inside the same site is compared to all entities of type B until a successful interaction occurs. The next type A object is then compared to all type B entities. When the entities have had chance to communicate, the next interaction rule is implemented. The UISS employs a set of fundamental features to mimic the conventional immune system apparatus and its reaction to a generic pathogen. This core, which is made up of entities, processes, and interactions, seldom changes unless novelties in biological knowledge are discovered and evaluated according to the above-mentioned Model and Transfer criteria. The set of fundamental features is periodically expanded to simulate new disorders. While the extensions may differ from one pathology to the next, the core remains the same.

### 2.2 TOX module in UISS

**Fig. 2:**
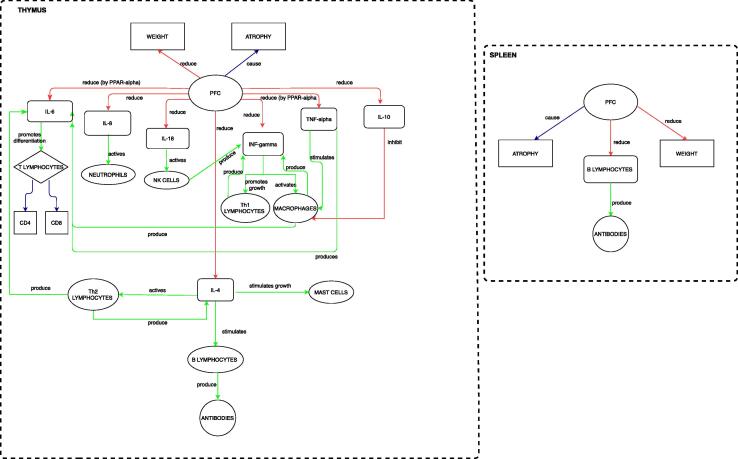
The PFAS – immune system interaction model. Conceptual description of the leading entities and interactions between PFAS and immune system. The main two compartments, the thymus and the spleen are depicted. The representation describes both cellular and humoral responses after exposure to PFAS.

To correctly reproduce the effects of PFOA/PFOS on the immune system and hence to create the physiology/disease layers in the UISS in silico trial computational platform, we retrieved from specialized literature all the entities, interactions and mechanisms of action that play a role in the context of use of interest. After a targeted and extensive search, we selected the papers that were best suited for this purpose. The final outcome of this phase was the development of the conceptual model that is depicted in Fig. 2.

In particular, it shows what we inserted into UISS to adapt it for the context of use, namely PFOS/PFOA-induced immunotoxicity. Specifically, after exposure to PFAS, thymus weight is reduced and there is an atrophy of the gland. PFAS can reduce the levels of many important cytokines, altering the activity of the immune system. Probably by a mechanism mediated by PPAR-α, PFAS reduce both IL-6 and TNF-alpha, but IL-4, IL-8, IL-18, IFN-γ and IL-10 levels also decrease.

IL-6 promotes differentiation of T lymphocytes into CD4 and CD8, hence PFAS cause an alteration of T-cell populations, especially CD4 and CD8. IL-6 is also produced by Th2 lymphocytes, activated by IL-4. IL-4 also decreases after PFAS exposure. As previously said, IL-4 activates Th2 lymphocytes but at the same time, it is produced by Th2 lymphocytes themselves. Moreover, IL-4 also stimulates the growth of mast cells, hence after PFAS exposure, a reduction (from the qualitative point of view) of these cells is expected. IL-4 also stimulates B lymphocytes to produce antibodies; in fact, one of the most important effects of PFAS on humoral response is the decrease of antibody levels.

PFAS cause a reduction of IL-8, which activates neutrophils, which have an important role in the immune response.

The decrease of IFN-γ level has a key role in the case of viral infections; IFN-γ is also produced by NK cells, which are activated by IL-18. So, PFAS act on IFN-γ by two means: reducing both IL-18 and IFN-γ itself. IFN-γ promotes the growth of Th1 lymphocytes and it activates macrophages, and both Th1 and macrophages can produce IFN-γ (through an autocrine mechanism). Macrophages also produce IL-6, which is one of the targets of PFAS.

The reduction of TNF-alpha levels is also relevant because this cytokine stimulates the macrophages and produces IL-6. PFAS can inhibit macrophage activity: by reducing both IFN-γ and TNF-alpha levels. However, on the other side, PFAS can stimulate the macrophage cells because these compounds consequently reduce IL-10 levels, which inhibits macrophages.

PFAS also affects the spleen: as in the thymus, PFAS cause atrophy of the spleen and reduce its weight. Here, one can notice their effects on the humoral response: PFAS reduce the level of B lymphocytes in the spleen, causing a decrease in antibody production.

## 3 Results

To establish the expectations of predictive accuracy for a computer modelling and simulation platform such as UISS-TOX, there is a need to assess its predictive ability. To this end, three different levels of observational depth can be envisaged:

● Level 1: we expect the model to predict any value among those observed within a reference population, or in a set of controlled experiments.
● Level 2: we expect the model to predict a median property (for example the average value) of the distribution of values observed in the reference population.
● Level 3: we expect the model to predict each of the values observed in the reference population or in the set of controlled experiments, when properly parametrised with subject-specific information.

To provide the most ambitious level of prediction i.e., level 3, UISS-TOX requires to be fed with a full “vector of features” i.e., specific input parameters that entirely personalize a patient. The complete list of input parameters is shown in Table 1.

**Table 1:** Vector of features showing the entire set of input parameters to obtain a fully personalized digital copy of the patient.

#	Model Input Description	UISS-TOX parameter name
1	PFOS in peripheral blood	PFOA
2	CD4 T cell type 1	Th1
3	CD4 T cell type 2	Th2
4	Macrophages	M
5	Dendritic cells	DC
6	Specific IgG titers	IgG
7	CD8 T cell	TC
8	Interleukin 1	IL1
9	Interleukin 2	IL2
10	Interleukin 10	IL10
11	Interleukin 12	IL12
12	Interleukin 17	IL17A
13	Interleukin 23	IL23
14	Type 1 IFN α (IFNA1)	IFN1A
15	Type 1 IFN β (IFNB1)	IFN1B
16	IFN-γ(*)	IFNG
17	TNF-α(*)	TNF
18	Vitamin D	VitaminD
19	FoxP3	Treg
20	Interleukin 10	IL10
21	TGF-β	TGFB
22	Age	Age
23	Body Mass Index	BMI
24	Disease model to be used	UISS-TOX-X (where X is the disease model module)
25	Vaccination	VC
26	Mechanism of Action of the Chemical	MoA (cellular and/or molecular)

As we were able to retrieve sufficient data to provide a level 2 validation, we skipped level 1. To this end, before starting the level 2 validation process, we preliminary tested the validity of UISS-TOX from the point of view of its capability to predict the physiological immune response to an initial challenge of chemicals. To this end, the following input experiment scenario was designed. The first step consisted of the generation of a virtual patient’s cohort. To generate an in silico patient, each feature must be assigned a single value. These values could be derived from individual physical patients; however, if a cohort of digital patients is to be created, a system for synthesizing as many diverse input vectors as required that are biologically/physiologically realistic should be in place. Formally, this necessitates characterizing the population's joint distribution of inputs. We have accumulated typical values and standard deviations for each feature, allowing us to create realistic values for each component individually. In this manner, the biological relationships between features would be ignored, and so a medically plausible input vector would not be guaranteed. As a result, to avoid medical inappropriateness, we must consider these relationships [90]. Two in silico cohorts of 100 virtual patients have then been generated according to that procedure, considering a properly functioning immune system, and an age ranging from 18 to 60 years old. The first in silico cohort (cA) was PFOA exposed at time 0 to reach a serum concentration of 10 ng/ml. The second one was not exposed (cB).

**Fig. 3:**
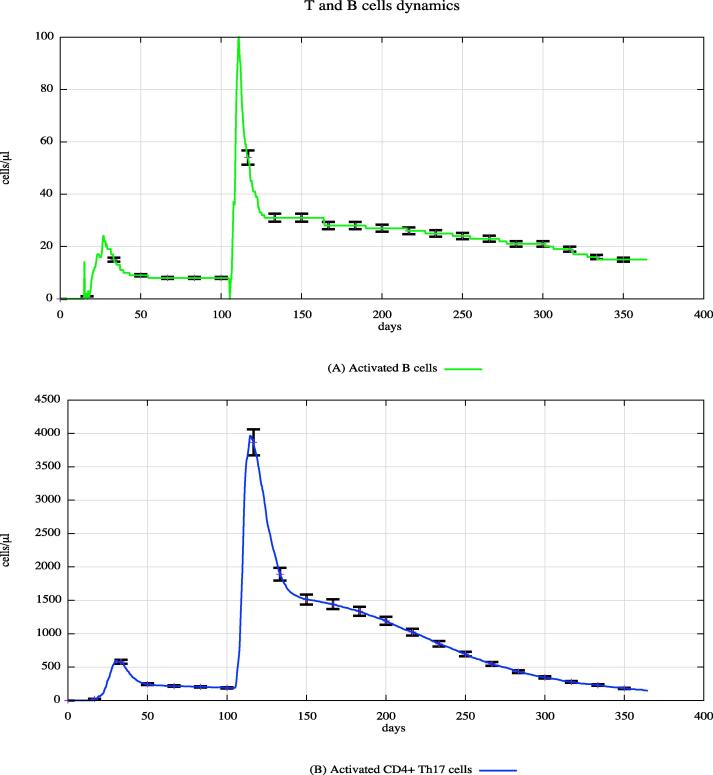
Population averaged immune system dynamics for the PFOA exposed in silico cohort at time 0. UISS-TOX in silico prediction of T (panel A) and B (panel B) cell dynamics are represented.

**Fig. 4:**
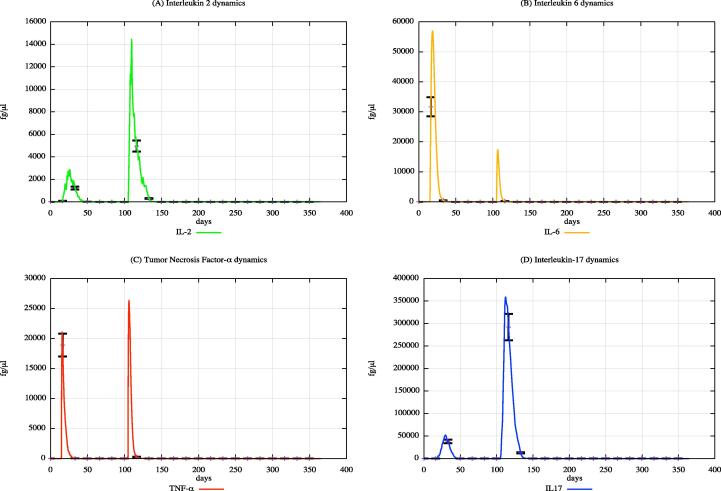
Population averaged immune system dynamics for the PFOA exposed in silico cohort at time 0. UISS-TOX in silico prediction of IL-2, IL-6, TNF-α and IL-17 dynamic levels are shown.

**Fig. 5:**
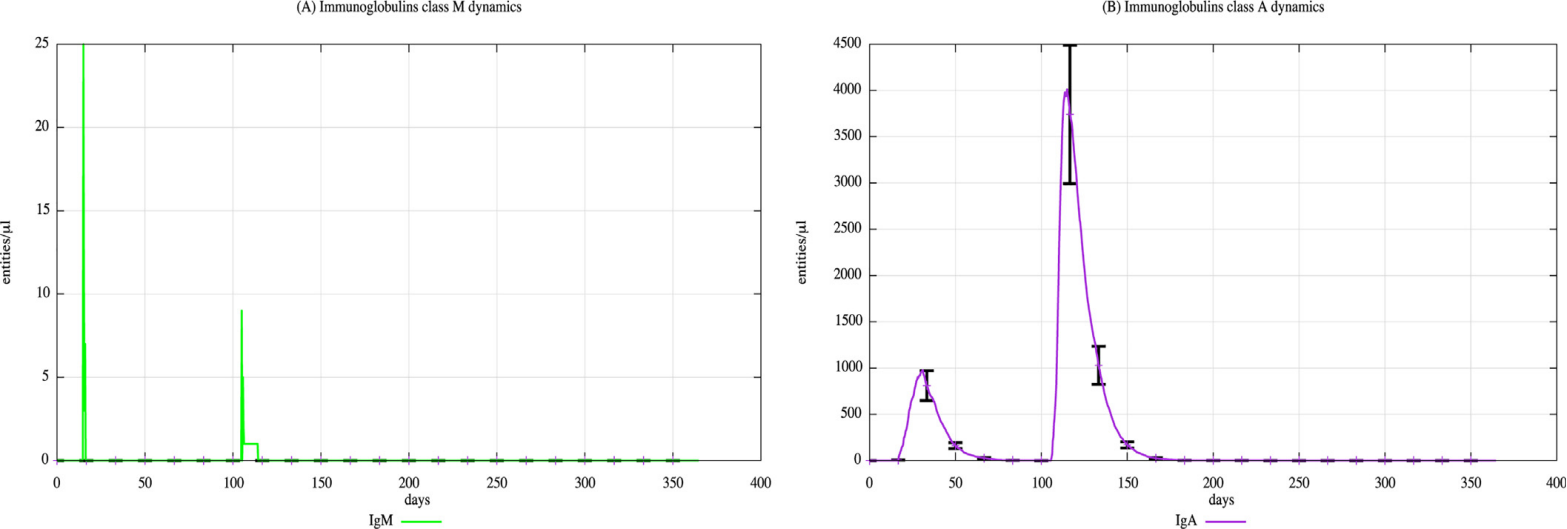
Population averaged immune system dynamics for the PFOA exposed in silico cohort at time 0. UISS-TOX in silico prediction of IgM and IgA dynamic levels are shown.

**Fig. 6:**
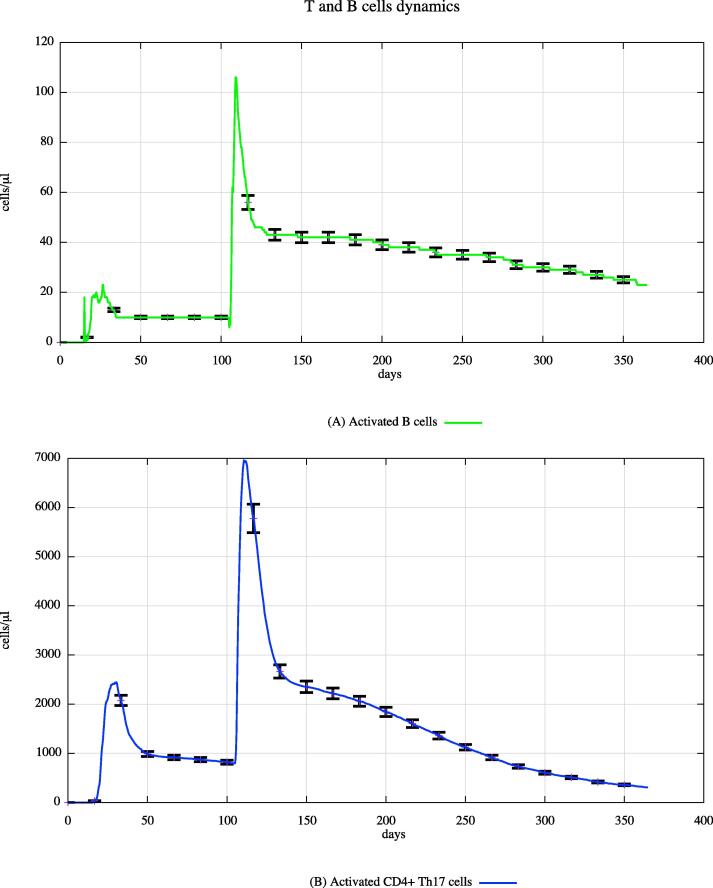
Population averaged immune system dynamics for the PFOA not exposed in silico cohort at time 0. UISS-TOX in silico prediction of T (panel A) and B (panel B) cell dynamics are represented.

**Fig. 7:**
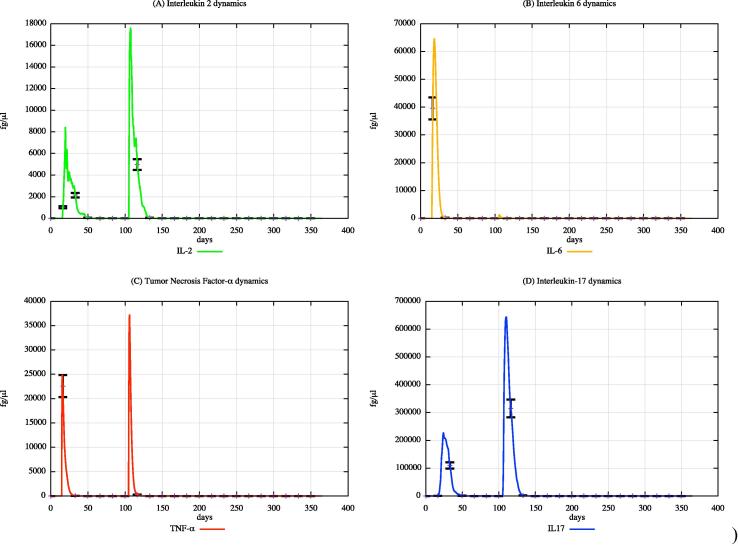
Population averaged immune system dynamics for the PFOA not exposed in silico cohort at time 0. UISS-TOX in silico prediction of IL-2, IL-6, TNF-α and IL-17 dynamic levels are shown.

**Fig. 8:**
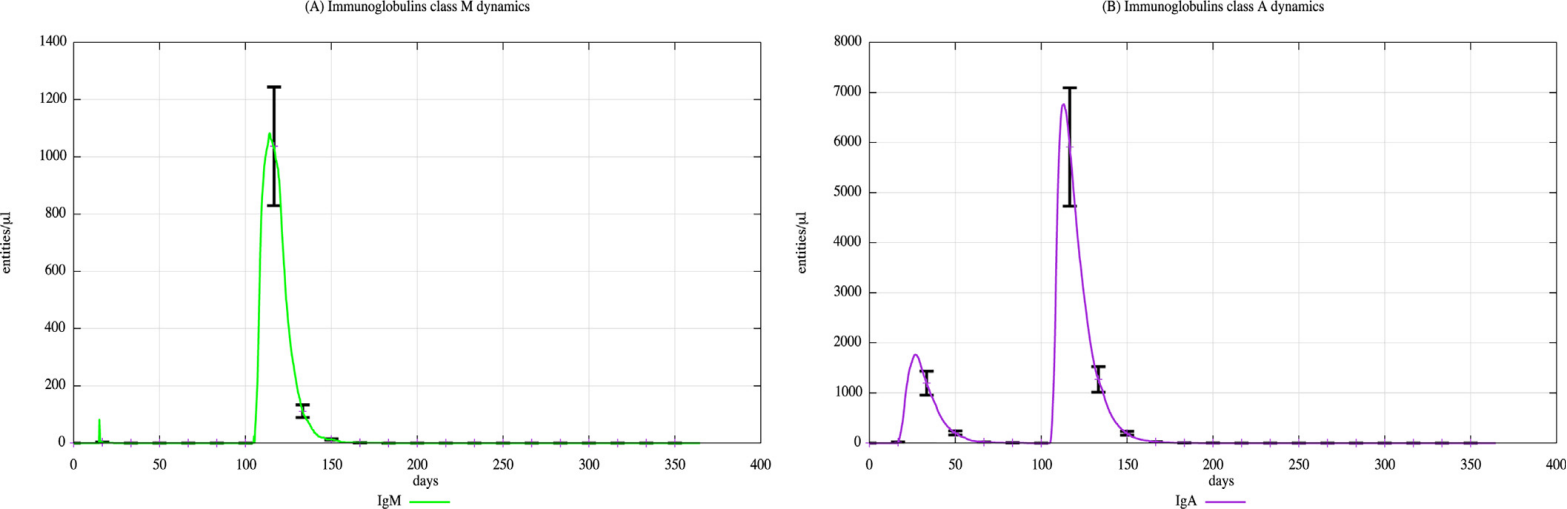
Population averaged immune system dynamics for the PFOA not exposed in silico cohort at time 0. UISS-TOX in silico prediction of IgM and IgA dynamic levels are shown.

In both cohorts, HLA-I and HLA-II were varied using a uniform distribution among Caucasian individuals. Each digital twin received two generic bacterial challenges (the first one at day 20 and the second one at day 100), to appreciate the effects of PFOA over the immune system responses. Fig. 3, Fig. 4, Fig. 5 show the population averaged immune system dynamics for cA, while Fig. 6, Fig. 7, Fig. 8 depict the population averaged immune system dynamics for cB. In particular, Fig. 3, Fig. 4, Fig. 5 show the well-known effects reported in the literature on immunoglobulins [91] and cytokines [92], as well as on T and B cell dynamics, illustrating the level 2 predictive capability of UISS-TOX.

From the comparison of the figures block 3–5 (cA, PFOA exposed) with the figures block 6–8 (cB, PFOA not exposed) one can appreciate that the peripheral blood serum concentration of 10 ng/ml of PFOA affects the immune system response both in cellular and humoral response.

To strength the level 2 validation of UISS-TOX, retrospective clinical data was gathered from different studies, showing pieces of evidence related to the effects elicited in the human immune system (especially in response to different vaccinations) by PFAS exposure [24], [26], [93].

**Fig. 9:**
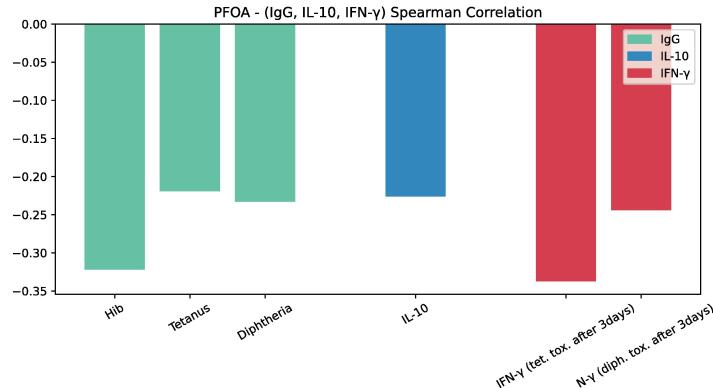
Inverse correlation between PFOA plasma concentration levels and vaccine antibodies against Hib, tetanus and diphtheria, as well as IFN-gamma production.

The first in silico experiment was designed to let UISS-TOX make predictions about the effects of PFOA elicited in anti-Hib, anti-Tetanus, anti-Diphtheria antibodies, IL-10 and IFN-gamma in children, like the ones observed in the study conducted by Abraham et al. [26]. We generated a cohort of in silico children patients (same numerosity of the real observational study) simulating a two-vaccination schedule. Fig. 9 shows a predicted inverse correlation between PFOA plasma concentration levels and vaccine antibodies against Hib, tetanus, and diphtheria, as well as IFN-γ production.

**Fig. 10:**
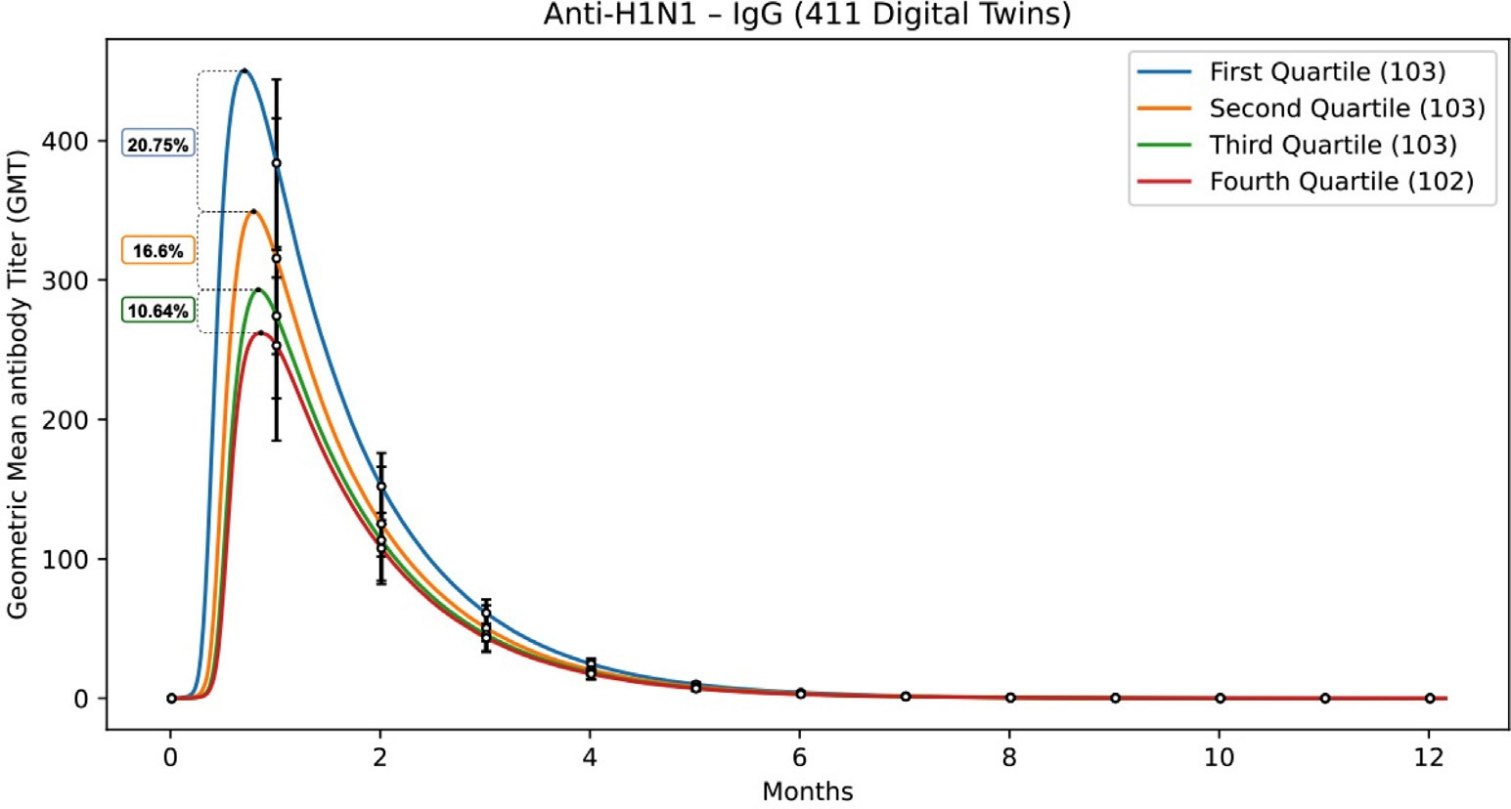
Predicted anti-H1N1 antibodies titers of in silico patient cohorts exposed to different PFOA concentrations (1st, 2nd, 3rd and 4th quartile) after having received influenza vaccination. These in silico predictions mirror the in vivo observed results depicted in Looker’s work.

The second in silico experiment compared UISS-TOX predictions against the data on anti-H1N1 antibodies titers presented in the study by Looker et al. A total of 411 digital twins were generated accordingly the inclusion criteria of the observational study. All generated in silico patients received the influenza vaccination. We then divided the virtual patients in 4 quartiles, depending on the PFOA concentrations; finally, we compared the predicted anti-H1N1 antibodies titers against the data presented in the paper by Looker et al. [93], obtaining results reported in Fig. 10 that are in good agreement with in vivo data.

**Fig. 11:**
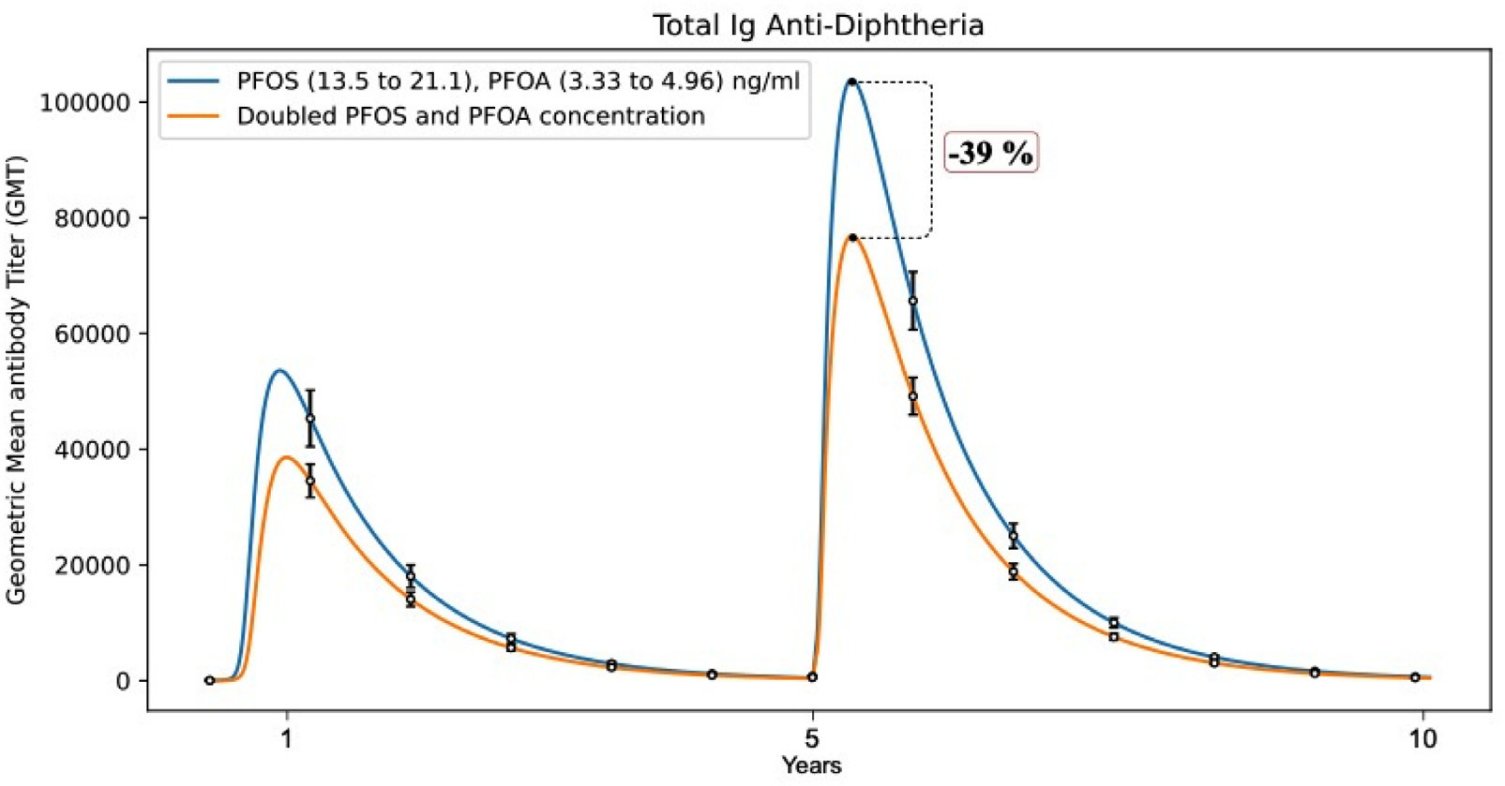
Predicted negative correlation with anti-Diphtheria antibodies titers of in silico children patient cohorts (at age of 5 years) exposed to different PFOS and PFOA concentrations after diphtheria immunization. These in silico predictions mirror the in vivo observed results depicted in Grandjean’s work, indicating that elevated exposures to perfluorinated compounds are associated with reduced humoral immune response to routine childhood immunizations.

Similarly, good agreement was found when simulating the third study by Grandjean et al. [24]. Fig. 11 shows how UISS-TOX is able to predict negative correlations with antibody concentrations at the age of 5 years, for which a 2-fold greater concentration of exposure was associated with a difference of −39% (95% CI, −55% to −17%) in the Diphtheria antibody concentration.

## 4 Discussion

Here, we describe how a mathematical model developed for a primary context of use (design of tuberculosis vaccines), for which it was developed and validated, can be repurposed and translated for a (“new”) target context of use (assessing interaction of the immune system with environmental contaminants, such as PFAS).

In Silico Trials are increasingly used for the assessment of biomedical products with the aim to reduce, refine, or replace in vitro, in vivo, or human experiments. Human experimentation is the most ambitious target for In Silico Trials, with increasing difficulty going from the refinement of clinical trials to their reduction, and ultimately to their replacement. A huge multidisciplinary effort is need to develop an in silico trial platform, especially from scratch. A considerable part of this effort concerns the retrieval, exploration and deep analysis of data provided by retrospective literature and/or experiments. A possible solution to reduce the time-consuming part in the in silico trial is to divide the modelling and simulation platform in three different, interconnected, levels i.e., the physiology model, the disease model and the treatment model. This allows all the efforts to be focused on a specific layer. Also, for the credibility assessment of the in silico trial platform, efficiencies are gained by incrementally establishing the validity of the computational platform. This “three-level” approach has another advantage - the possibility to explore a context-switching from one disease to another, while keeping the physiology layer.

In the present work the focus was on the interaction of chemicals with the immune system, but it must be considered that there are processes that are not taken into account with the present model, such as ADME/kinetic processes, and understanding PK profiles of a chemical.

In particular, we focused on PFAS, as they represent an environmental relevant class of compounds of high concern. PFOA and PFOS have been selected as reference PFAS, as they are two of the most widely used and studied chemicals in the PFAS group, with many human data available. Based on published data, and the understanding of the underlying mechanisms of PFAS-induced immunotoxicity, it was possible to feed the model with immunological parameters altered by PFOA or PFAS, and in return, the model was able to correctly predict the reduced antibody production observed in humans. Several studies document that PFAS exposure is associated with suppression in at least one measure of the anti-vaccine antibody response with evidence from developmental, childhood, and adult exposures [22], therefore, as immunological parameter, the response to vaccination was chosen. UISS-TOX was able to reproduce such data.

An immediate use of the UISS-TOX could be to estimate the benchmark dose, the dose or concentration that produces a predetermined change in the response rate of an adverse effect, for example 5 or 10% reduction in antibody response [94]. Conversely, we can ask the model to estimate the plasma concentration of PFOA/PFOS associated with a 5 or 10% decrease in antibody production.

Another use of UISS-TOX, could be to estimate the reduced response to vaccination in a population or at the individual level based on PFOA/PFAS plasma levels, e.g., in a polluted area. This may help the health authorities/policy makers to estimate, for example, the possible cases of infections, due to a lack of vaccine protection, and the related costs in a PFAS-contaminated area.

UISS-TOX may also be used to predict the immunotoxic potential of other PFAS. Considering the hundreds of PFAS used, alternative approaches to the use of the animals are essential to investigate their immunotoxicity. As no in vivo or in vitro data are available for the majority of PFAS, we can imagine to perform ad hoc in vitro studies to obtain useful data to be input to the model, e.g., cytokine production, effects on lymphocyte subpopulations (see parameters listed in Table 1), to obtain a prediction of antibody production. Experiments are ongoing to verify this working hypothesis. Similarly, other classes of compounds, for which in vivo data are limited to a few compounds, represent the ideal situation to take advantage of the UISS-TOX.

A potential limitation of our approach is represented by the difficulties in predicting the immune response to an unknown substance. However, the computational framework is able to take advantage from in vitro experiments. Hence, considering that UISS-TOX includes all the main cells involved in the specific immune response, it is conceivable that by knowing the biological processes modified by any immunotoxic substance, the corresponding input parameter can be easily inserted into the model, which as output will tell whether or not there is a risk of reduced response to vaccination.

We have demonstrated that the UISS-TOX may offer the opportunity to estimate the immunotoxicity risk posed by PFAS. Thus, the integration of in vitro methods and computer simulations provides a means of establishing new knowledge and approaches to protect health from potential immunotoxicants, while also avoiding in vivo experiments.

To make the presented model strategy more robust, the applicability of UISS-TOX will also be tested in a follow-up project using bisphenols and skin sensitizing chemicals, considering that the immune system response, especially in animals, is the most sensitive indicator of dioxin toxicity (TCCD, for example).

Finally, the interoperability of the UISS-TOX with Physiological Based Kinetic models should be explored as an integrated approach in modelling the mode of action of chemicals from exposure to effect, allowing for different time and spatial scales.

## CRediT authorship contribution statement

**Francesco Pappalardo:** Conceptualization, Supervision, Methodology, Software, Writing – original draft. **Giulia Russo:** Validation, Data curation, Writing – original draft, Writing – review & editing. **Emanuela Corsini:** Resources, Writing – review & editing, Formal analysis. **Alicia Paini:** Resources, Writing – review & editing, Funding acquisition. **Andrew Worth:** Writing – review & editing, Funding acquisition, Project administration.

## Declaration of Competing Interest

The authors declare that they have no known competing financial interests or personal relationships that could have appeared to influence the work reported in this paper.

## Footnotes

6 https://www.oecd.org/officialdocuments/publicdisplaydocumentpdf/?cote=ENV-JM-MONO(2018)7&doclanguage=en).
